# Comparison of the remineralization effectiveness of three remineralizing agents on artificial enamel lesions: an in vitro study

**DOI:** 10.1038/s41405-025-00330-y

**Published:** 2025-05-05

**Authors:** Maha Mohamed Montaser, Heba Youssef, Ghada Mohamed Mahmoud

**Affiliations:** 1 The Arab Academy for Science, Technology and Maritime Transport- College of Dentistry-Alamein Campus, Alexandria, Egypt; 2 The pediatric Dentistry department at the Arab Academy for Science, Technology and Maritime Transport- College of Dentistry-Alamein Campus, Alexandria, Egypt

**Keywords:** Health sciences, Diseases, Salivary gland diseases

## Abstract

**Introduction:**

Early enamel demineralization can be reversed through remineralization, which restores lost minerals to strengthen enamel and prevent decay.

**Aim:**

This study evaluated the remineralization efficiency of three commercial treatments on artificially demineralized primary enamel.

**Methods:**

Forty exfoliated primary anterior teeth were demineralized and divided into five groups: untreated control, artificial saliva, fluoridated toothpaste, Curasept toothpaste, and BioMin toothpaste. The treatments were applied for 28 days. Remineralization efficacy was assessed using Vickers microhardness testing, surface roughness measurement, and Scanning electron microscope combined with EDX (SEM-EDX). One-way ANOVA and Tukey’s HSD test were used for statistical analysis.

**Results:**

Microhardness and surface roughness tests confirmed BioMin’s superior remineralization potential. Scanning electron microscopy showed that untreated enamel exhibited extensive demineralization, whereas treated groups displayed varying degrees of remineralization. BioMin demonstrated the highest calcium, phosphate, and fluoride incorporation, followed by Curasept and fluoridated toothpaste. The artificial saliva group showed no significant improvement over the control.

**Conclusion:**

BioMin, followed by Curasept and fluoridated toothpaste, effectively remineralized demineralized enamel. BioMin’s bioactive glass formulation provided the highest mineral gain, suggesting its potential for non-invasive enamel restoration in pediatric dentistry.

## Background

Caries is the most prominent long-term illness, and it continues to be a major challenge in dentistry [1]. This condition is characterized by a biofilm-driven, diet-influenced, and changeable process that leads to the net loss of minerals [2]. The current understanding of caries development centers around the repeated cycles of removal and addition of minerals, triggered by acid-producing microorganisms in the oral cavity [3, 4].

Tooth decay is largely preventable and can be overturned in its primary stages. The demineralization of enamel and dentin can be stopped by preventing biofilm formation and leveraging the defending components in saliva. Recently, caries research has increasingly focused on developing conservative ways to manage enamel demineralization [5].

Enamel remineralization is the process by which essential minerals, such as calcium and phosphate, are redeposited into the tooth enamel, helping to repair the early stages of demineralization and strengthen the enamel against future acid attacks [6].

The DEM-REM (demineralization-remineralization) cycle is continuous and affected by various factors, including the pH of saliva. Saliva helps neutralize plaque acids and supplies minerals to the enamel. However, the gradual remineralization process through saliva does not lead to a net mineral gain [6–8].

Numerous studies indicate that fluoride (F) remains the most effective agent for promoting remineralization. This effectiveness is due to its enhanced precipitation of Ca2 + /PO4-3 into the tooth enamel to form fluorapatite crystals (Ca10(PO4)6F2). *Fluorapatite* is a naturally occurring mineral and a key component of tooth enamel. It is a more acid-resistant form of *apatite*, the mineral that makes up the hard outer layer of teeth and bones. Fluorapatite is formed when fluoride ions replace some hydroxyl ions in hydroxyapatite, the mineral that makes up tooth enamel [9].

While fluoride has long been a cornerstone in restoring initial carious lesions and has proven to decrease the severity of dental caries in children, there has recently been a decreased acceptance among the public in both home-use formulas with their risk of systemic toxicity, and professional formulas with high concentrations [10, 11]. There is a need for an effective alternative that can be used at home with high remineralizing potential and a wider safety margin. Hydroxyapatite-containing toothpastes have recently been shown to be effective as remineralizing agents [12–15].

Incorporating remineralizing toothpaste into the preventive care of children potentially decreases the DMF of permanent and primary teeth, thus decreasing the number of dentist visits and increasing oral health in young children. The evidence for this regimen was provided by several research and review articles [15–19].

Curasept toothpaste consists of amorphous calcium phosphate nanoparticles enhanced with fluoride. This complex provides essential calcium and phosphate ions for the formation and repair of tooth enamel, helping to fill in micro-pores or holes. The fluoride(1450 ppm) in the formula improves enamel resistance to acid attacks and supports remineralization, forming fluorapatite, a more acid-resistant enamel. The non-abrasive nanoparticles make it especially beneficial for individuals with dental sensitivity [20].

BioMin products use bioactive glass technology. Compared to Novamin (the first generation of bioactive glass), BioMin F is superior as it contains both fluoride and optimal calcium and phosphate proportions. When the oral pH drops after consuming acidic or sugary food, BioMin’s bioactive glass dissolves faster, releasing minerals and fluoride to neutralize acid and stabilize pH, protecting teeth from decay. The product provides a prolonged protective effect, slowly releasing ions over time, and forms a stable protective layer on the enamel that lasts for hours, unlike regular toothpaste. Biomin for kids contains a lower fluoride content (530 ppm), So the risk of fluorosis is minimized to be safer for children while still offering 12-hour fluoride protection [21].

Both F-ACP (Curasept 1450ppm) and the new bioactive glass (Biomin (1450 ppm) have been previously studied for reducing dentine hypersensitivity by occluding dentinal tubules. This study fills the gap in the literature by evaluating the effects of F-ACP (Curasept 1450 ppm) and bioactive glass (Biomin 580 ppm) on the enamel of primary teeth over an extended period. Enamel of primary teeth is thinner and contains less mineral content than permanent teeth, so it is more susceptible to demineralization and harder to remineralize. The study focuses on the use of Biomin (580 ppm), which is considered safer for children, to assess its effectiveness in remineralizing primary tooth enamel. Examples from previous studies: Vitiello F. et al. (2023) [9] worked on remineralizing extracted permanent molars over 7 days using Curasept(1450ppm) while Eldeeb AI (2024) [22] utilized Biomin toothpaste (1450ppm) to remineralize extracted premolars over 2 weeks.

Pediatric dentists can use this information to tailor remineralization treatments based on a child’s specific needs—whether it be in response to early enamel demineralization, sensitivity, or prevention of further decay. This aligns with current trends toward preventive care, making an important contribution to non-invasive treatment options in pediatric dentistry.

Therefore, our purpose was to investigate and relate the remineralizing efficiency of three different commercially available treatments on fake human enamel lesions in primary teeth by using Biomin kids with lower fluoride content (580 ppm), Curasept (1450 ppm) and conventional toothpaste (1450ppm) after 28 days of treatment, using SEM combined with EDS, surface roughness and microhardness tests. The proposed null hypothesis stated that there are statistically no significant differences in Vickers hardness number and surface roughness values among the tested groups or agents, nor the Scanning electron microscope combined with EDX (SEM-EDX) results.

## Materials and methods

This study received approval from the ethics committee at the AASTMT Alamein campus Faculty of Dentistry with a code 301/2024 [9].

### Sample size estimation

The sample size was estimated using Power Analysis and Sample Size Software (PASS 2020), assuming an effect size of 20%, a significance level of 5%, and a power of 80%. The methodology follows Muralidharan’s (2014) and Vitiello et al. (2022) recommendations, ensuring statistical robustness. A minimal total of 40 samples (8 per group) was sufficient for comparing the effectiveness of different remineralizing agents on artificial enamel lesions. The sample size calculation accounted for expected variations in mineral content changes post-treatment and was validated using a chi-square test to ensure adequate power for detecting statistically significant differences among groups [8, 23].

### Randomization process

Forty sound human primary anterior teeth were obtained from the Department of Pediatric Dentistry and Dental Public Health clinic at the AASTMT Faculty of Dentistry. A visual examination ensured that the teeth met the inclusion criteria, confirming they were free from caries, previous fillings, developmental anomalies, and cracks under magnification. Random allocation was implemented using a computer-generated randomization method to assign samples into groups, minimizing selection bias and ensuring an even distribution of variables.

This study described measures taken to minimize bias, such as blind assessments, random allocation, standardized sample handling, and calibrated instrumentation. The evaluators conducting enamel remineralization assessments were blinded to the treatment groups to prevent subjective bias. A computer-generated randomization method was used to evenly distribute samples across groups, minimizing selection bias. All samples underwent identical handling procedures, including controlled environmental factors, pH cycling, and artificial saliva storage. Additionally, all instruments, including the microhardness tester and SEM-EDS, were regularly calibrated to ensure consistent and accurate measurements across all samples.

### Instrumentation calibration

The microhardness tester was calibrated before each measurement session using a certified reference material with a known hardness value. Calibration involved adjusting the applied load, verifying the indentation depth, and conducting periodic revalidation using standard hardness blocks. Additionally, the device was checked at regular intervals to detect and correct any deviations, ensuring precision and consistency. The SEM-EDS system was also calibrated using a standard reference material for elemental analysis, ensuring accurate measurement of calcium, phosphorus, and fluoride levels in the enamel samples. This calibration process follows established protocols outlined in ISO 6507-2:2018 for microhardness testing and ASTM E1508 for EDS calibration.

### Sample preparation

The enamels were washed with fluoride-free abrasive, cleaned using purified water, and dried by air. A 3 × 4 mm square was formed above the CEJ (cemento-enamel junction) on the facial aspect. Acid-proof nail polish was painted on all of the teeth’s surfaces [4]. After the nail polish evaporated the self-sticking strips were removed, leaving a 3 × 4 mm exposed enamel window in the middle third of the labial surface, then fixed with an acrylic resin that was self-cured with their labial surfaces orientated upward for simple handling [24–26].

### Grouping and methods

The samples were split into five groups as follows: (*n* = 8 in each group).

Group 1: demineralized group with no treatment.

Group 2: Group stored in artificial saliva.

Group 3: Fluoridated toothpaste (Signal 2) Group treated with conventional toothpaste with pH=7.1.

Group 4: treated with Curasept toothpaste with pH = 10.05.

Group 5: (Biomin Group): treated using Biomin toothpaste with pH = 10.76.

### Preparation of the demineralizing solution and artificial saliva

A demineralizing solution was prepared to create artificial lesions, containing 2.2 mM CaCl2, 2.2 mM KH2PO4, and 0.05 M acetic acid, with the pH adjusted to 4.4 using 1 M KOH. The artificial saliva comprised 1.5 mM CaCl2, 0.9 mM NaH2PO4, and 0.15 M KCl, with a pH of 7 [26].

### Lesion formation

Forty primary teeth were scheduled for extraction due to pre-shedding mobility and were gathered and stored in a 0.2% thymol solution. After sample preparation as described in section “Sample preparation”, the teeth were immersed in a demineralizing solution for 96 h and the damaged specimens were discarded [26].

### Remineralization protocol through pH cycle modeling

The cyclic regimen included a 2 h daily acid challenge in demineralizing solution (DS). The remineralization treatment was applied to the demineralized areas using a micro brush in circular motions twice a day (morning and night), each application lasting two minutes. For the remaining 24 h, the samples were kept in fresh artificial saliva (AS). In group 2, the samples were immersed in AS for the entire study. The AS was prepared daily and stirred magnetically at 350 rpm, while the DS treatment remained static. The DS treatments were conducted at 37 °C in an incubator, with the rest of the experiment taking place at room temperature (Table 1).

**Table 1:** Chemical structure of the examined products [9, 43, 44].

material	manufacturer	Ingredients
Fluoridated toothpaste (Signal 2)	Unilever, Egypt	Sodium Monofluorophosphate (1450 ppm fluoride), calcium carbonate, Aqua, Sorbitol, Hydrated Silica, Sodium Lauryl Sulfate, Sodium Monofluorophosphate, Aroma, Potassium citrate, Cellulose Gum, Trisodium phosphate, Sodium Saccharin, Phenylcarbinol, Zinc oxide, Limonene.
Biomin	Queen Mary University of London, United Kingdom	Glycerin, Silica, PEG 400, Fluoro Calcium Phospho Silicate, Sodium Lauryl Sulphate, Aroma, Carbomer, Potassium, Acesulfame, Fluoride 580 ppm.
Curasept Biosmalto	Curasepta Spa, Italy	Nanoparticles of Amorphous calcium phosphate enriched with carbinate and fluoride, citrate outer layer, fluorohydroxyapatite (1450 ppm fluoride), purified water, Hydrated silica, MgSrCarbinate, Hydroxyapatite conjucated with chitosan, Cellulose gum, Xylitol, Cocamidopropyl betaine, Sroma, Sodium Monofluorophosphate, Xantham gum, Potassiumacesulfame, Ethylexylglycerin, Phenoxyethanol, Sodium benzoate, Citric acid.

pH cycle: 7 am brushing (2 min) then kept in AS →soaked in demineralized solution for 2 h(12pm–2pm) then kept in AS→7 pm brushing(2 min) then kept in AS for the next day

The experiment lasted for 28 days, and upon completion, the blocks were assessed using SEM, EDX, microhardness, and surface roughness analysis [27].

### Surface roughness

The specimens’ Ra values were determined using an optical profilometer (MARSURF PS1, Mahr GmbH Göttingen, Göttingen, Germany). Each specimen had four tracings perpendicular to the surface, and the average of them was analyzed to calculate the final Ra score [28].

### Microhardness test

The enamel of each tooth in all groups was assessed using a Vickers microhardness instrument (Wilson microhardness tester, Japan) with a 25 g force for 5 s. Three points were measured on each sample, and their average was calculated. Microhardness assessment occurred at two key points: after the initial formation of caries (first assessment). and after the study (second assessment) [29].

### Scanning electron microscope analysis

The specimens were dried in the air, mounted on aluminum stubs, and then examined by a JEOL–JSM-IT 200 scanning electron microscope (Faculty of Science, Alexanderia University, Alexandria Egypt). Images were needed to test enamel topography through 500, 1000, 2000, 3000, and 10,000 magnifications. EDS analysis was investigated in 3 different areas for each tooth with those parameters: Operating distance: 15 mm, activation voltage: 25 kV, magnification: 500 [22].

### Statistical analysis

Data was processed using SPSS by IBM for Windows operating system 23.0. The Shapiro-Wilk test was used to check for normality, and all variables showed a normal distribution. Consequently, the means and standard deviations (SDs) were calculated. The EDS, microhardness, and surface roughness results were examined using statistical comparisons between groups using one-way ANOVA (analysis of variance) followed by Tukey’s test. The group size was set to *n* = 8 for all experimental groups, and significance was set at *p* < 0.05 [22].

## Results

### Mineral content (Ca, P, F) using EDX

Tables 2, 3, and 4 present the average percentage by mass of mineral content (calcium, phosphate, and fluoride) determined through elemental analysis using EDX for each tested group.

**Table 2:** %Mass of calcium level at various points in the enamel surface of all studied groups.

	Demineralized (Group 1)	Saliva (Group 2)	Fluoridated toothpaste (Group 3)		Curasept (Group 4)	Bio-Min toothpaste (Group 5)
Mean ± SD	16.285±0.97	18.03625±3.27	25.77875±2.58		26.145±2	31.07375±3.3
One-way ANOVA test (*P* value)	*p* < 0.001*					
Tukey’s HSD Test (*P* value)						*P* < 0.001*
	*p* = 0.3639		*p* = 0.8485			
Min-Max	15–18	18.9–22.8	20.7–29.14		23–28.5	26.3–36.4

*HSD* Honestly Significant Difference, *SD* standard deviation.

* significant (*p* < 0.05).

**Table 3:** %Mass of phosphorous level at various points in the enamel surface of all studied groups.

	Demineralized (Group 1)	Saliva (Group 2)	Fluoridated toothpaste (Group 3)		Curasept (Group 4)	Bio-Min toothpaste (Group 5)
Mean ± SD	9.905 ± 0.69	9.3525 ± 1.80	12.86125 ± 1.88		14.2175 ± 0.25	16.0475 ± 0.38
One-way ANOVA test (*P* value)	*p* < 0.001*					
Tukey’s HSD Test (*P* value)						*P* < 0.001*
	*p* = 0.4438		*P* = 0.0710			
Min-Max	9–11	18.9–22.8	11–15		14–14.69	15.5–16.5

*HSD* Honestly Significant Difference, *SD* standard deviation.

* significant (*p* < 0.05).

**Table 4:** %Mass of fluoride level at various points in the enamel surface of all studied groups

	Demineralized (Group 1)	Saliva (Group 2)	Fluoridated toothpaste (Group 3)		Curasept (Group 4)	Bio-Min toothpaste (Group 5)
Mean ± SD	0.15 ± 0.17	0.17125 ± 0.169	0.69625 ± 0.16		0.7775 ± 0.33	1.09875 ± 0.43
One-way ANOVA test (*P* value)	*p* < 0.001*					
Tukey’s HSD Test (*P* value)						*P* < 0.001*
	*P* = 0.8633		*P* = 0.5066			
Min-Max	0.01-0.49	0.01-0.5	0.5-0.99		0.5-1.45	0.56-1.5

*HSD* Honestly Significant Difference, *SD* standard deviation.

* significant (*p* < 0.05).

To analyze the data, a one-way ANOVA was conducted to determine if there were significant differences between the groups for the percentages of calcium (Ca), phosphorus (P), and fluoride (F). Following the ANOVA, Tukey’s HSD (Honestly Significant Difference) test was used as a post-hoc analysis to identify which specific groups differed from each other. Comparing the Ca, P, and F percentages revealed that Groups 1 and 2 significantly differed from Groups 3, 4, and 5. However, no significant difference was found between Groups 1 and 2 in calcium, phosphorus, and fluoride percentages (*p* = 0.3639, 0.4438, and 0.8633, respectively). Furthermore, Group 5 showed the most significant differences compared to other groups. No significant difference was observed between Groups 3 and 4 in the percentages of calcium, phosphorus, and fluoride (*p* = 0.8485, 0.0710, and 0.5066, respectively).

### The surface microhardness

Table 5 presents the average microhardness determined through Vickers test for each tested group.

**Table 5:** Vickers the microhardness (VHN) readings for all groups

	Demineralized (Group 1)	Saliva (Group 2)	Fluoridated toothpaste (Group 3)	Curasept (Group 4)	Bio-Min toothpaste (Group 5)
Mean ± SD	77.91 ± 32.84	146.10 ± 44.11	169.98 ± 81.65	227.59 ± 44.82	284.05 ± 33.33
Tukey’s HSD Test (*P* value)	*P* < 0.001*	*P* = 0.598		*P* < 0.001*	*P* < 0.001*
Min-Max	31.13-138.21	69.6-221.7	22.9-283.3	146.65-298.90	196.8-360

*HSD* Honestly Significant Difference, *SD* standard deviation.

* significant (*p* < 0.05).

To analyze the data, a one-way ANOVA was conducted to determine if there were significant differences between the groups for the microhardness. Following the ANOVA, Tukey’s HSD (Honestly Significant Difference) test was used to identify which specific groups differed from each other. The descriptive statistics for the VHN data of the five test groups— group 1, 2, 3, 4, 5- were (77.91 ± 32.84), (146 ± 44.11), (169.98 ± 81.65), (227.59 ± 44.82), and (284.05 ± 33.33) respectively. One-way ANOVA yielded a P-value significantly less than 0.05, showing statistically significant differences in VHN among the five groups. The Tukey HSD test reveals statistically significant differences in VHN values between most treatment groups (*p* < 0.05), except between group 1 “AS VHN” and group 2“Signal 2 VHN.” This suggests that most remineralization treatments have distinct impacts on enamel hardness, with group 1 and group 2 being the most similar in their effects.

### The surface roughness

Table 6 presents the average surface roughness determined through optical profilometer (MARSURF PS1, Mahr GmbH Göttingen, Göttingen, Germany) for each tested group.

**Table 6:** Surface roughness (Ra) readings for all groups.

	Demineralized (Group 1)	Saliva (Group 2)	Fluoridated toothpaste (Group 3)	Curasept (Group 4)	Bio-Min toothpaste (Group 5)
Mean ± SD	2.484 ± 0.256	1.544 ± 0.190	1.701 ± 0.291	1.182 ± 0.291	0.937 ± 0.315
Tukey’s HSD Test (*P* value)	*P* < 0.001*	*P* = 0.266		*P* < 0.001*	*P* < 0.001*
Min-Max	2.003-2.921	1.181-1.827	1.230-2.219	0.662-1.766	0.490-1.419

*HSD* Honestly Significant Difference, *SD* standard deviation.

* significant (*p* < 0.05).

To analyze the data, a one-way ANOVA was conducted to determine if there were significant differences between the groups for the surface roughness. Following the ANOVA, Tukey’s HSD (Honestly Significant Difference) test was used to identify which specific groups differed from each other. The descriptive statistics for the VHN data of the five test groups— group 1,2,3,4,5were (2.484 ± 0.256), (1.544 ± 0.190), (1.701 ± 0.291), (1.182 ± 0.291) and (0.937 ± 0.315) respectively. One-way ANOVA yielded a P-value significantly less than 0.05, showing statistically significant differences in Ra among the five groups. The Tukey HSD test reveals statistically significant differences in Ra values between most treatment groups (*p* < 0.05), except between group 1 “AS Ra “ and group 2 “Signal 2 Ra.” This suggests that most remineralization treatments have distinct impacts on enamel roughness, with “AS Ra” and “Signal 2 Ra” being the most similar in their effects.

**Fig. 1:**
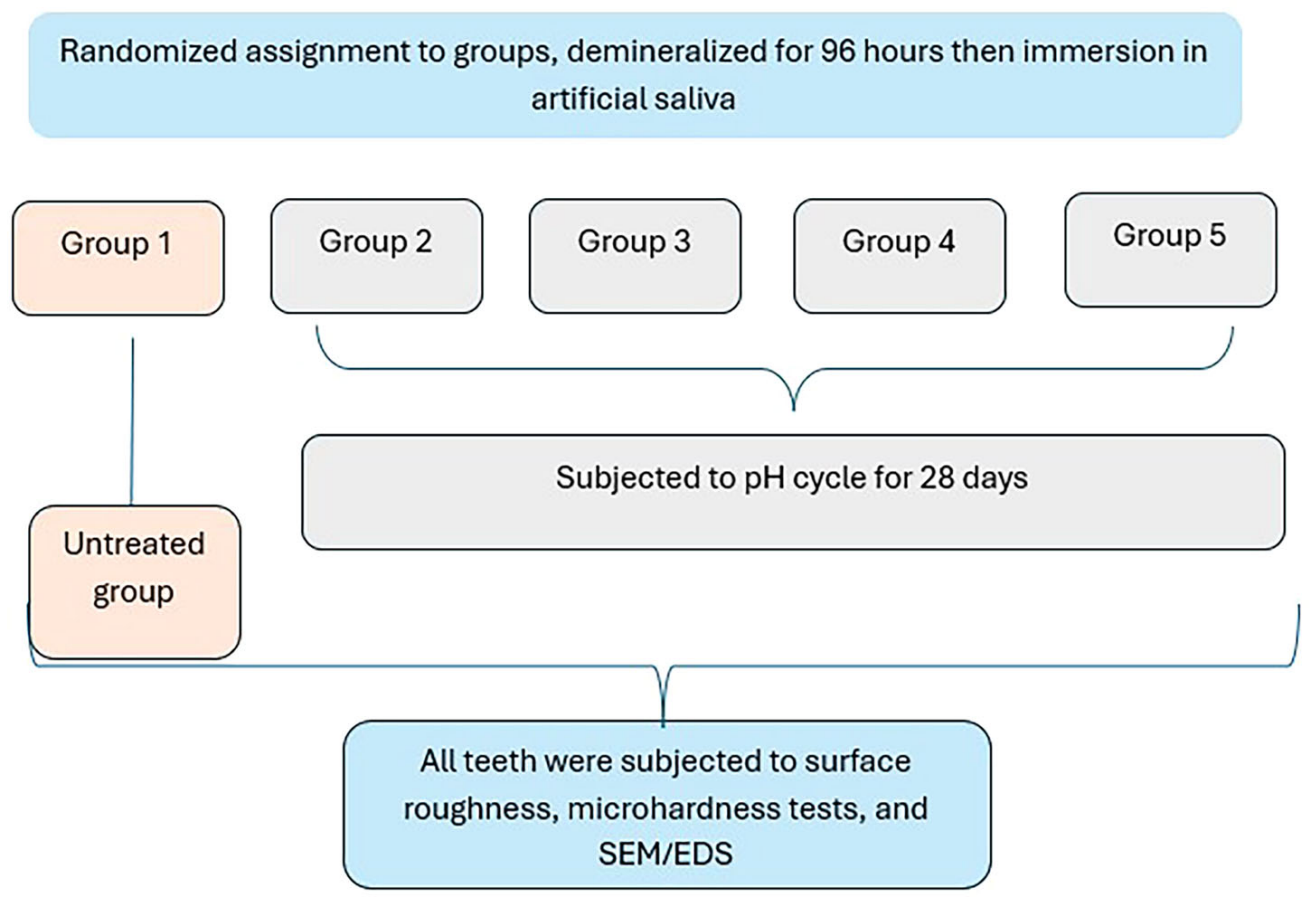
The outline illustrates important points in the experimental process. This design shows: G1 (untreated group), G2 (saliva remineralized Group), G3 (Signal toothpaste), G4 (Curasept Group), G5 (BioMin Group).

**Fig. 2:**
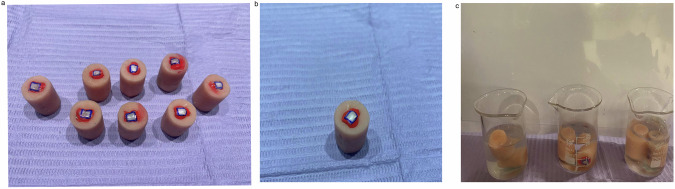
Steps of teeth preparation. **a** At the base of an acrylic block, each tooth is identified by a number. **b** A 4 x 3 mm window in the middle part of the tooth’s labial surface. **c** Each group was kept in a container filled with artificial saliva and labeled with the group name.

**Fig. 3:**
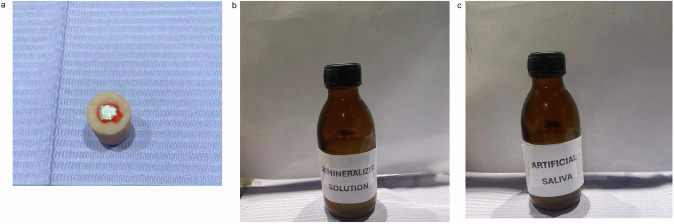
Items used in the experiment. **a** White spot lesions formed following contact to a demineralizing solvent. **b** demineralization solution. **c** Artificial saliva.

**Fig. 4:**
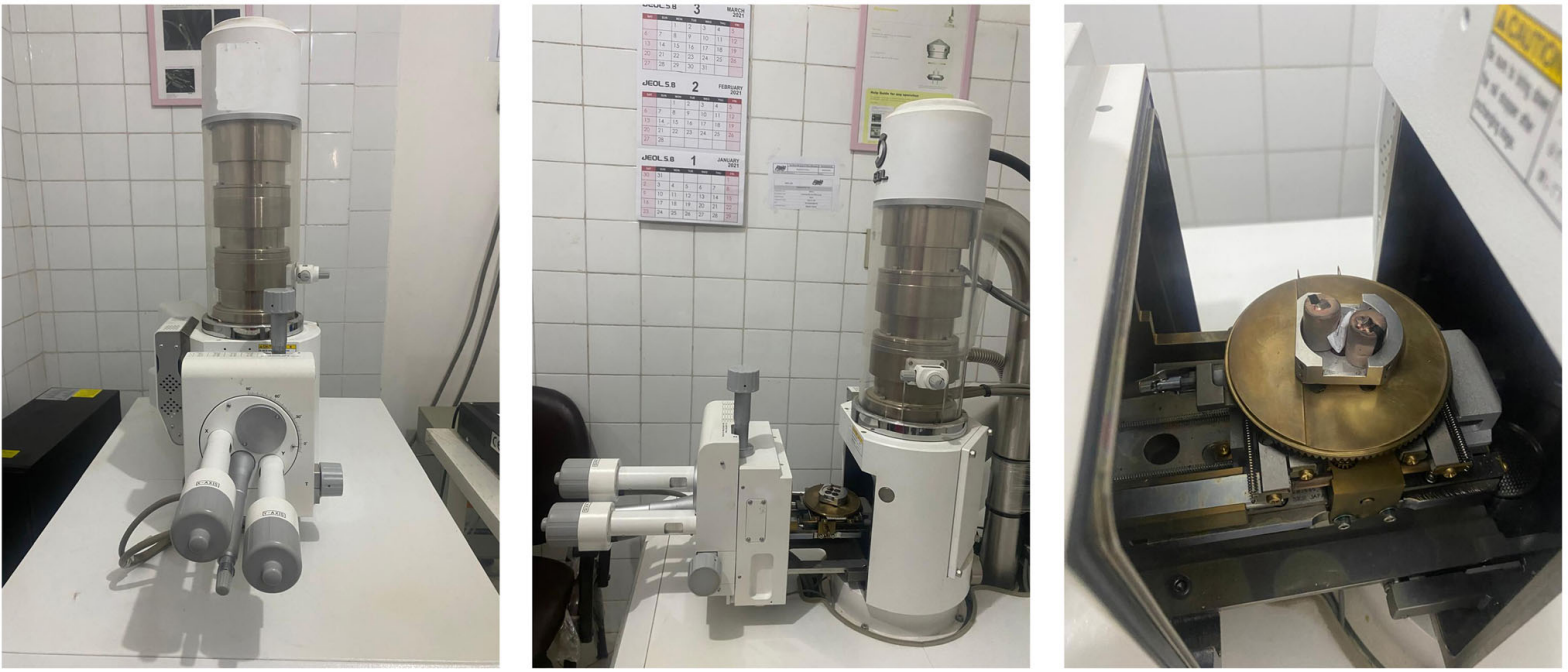
Different views of the scanning electron microscope. Scanning electron microscope attached with EDX unit.

**Fig. 5:**
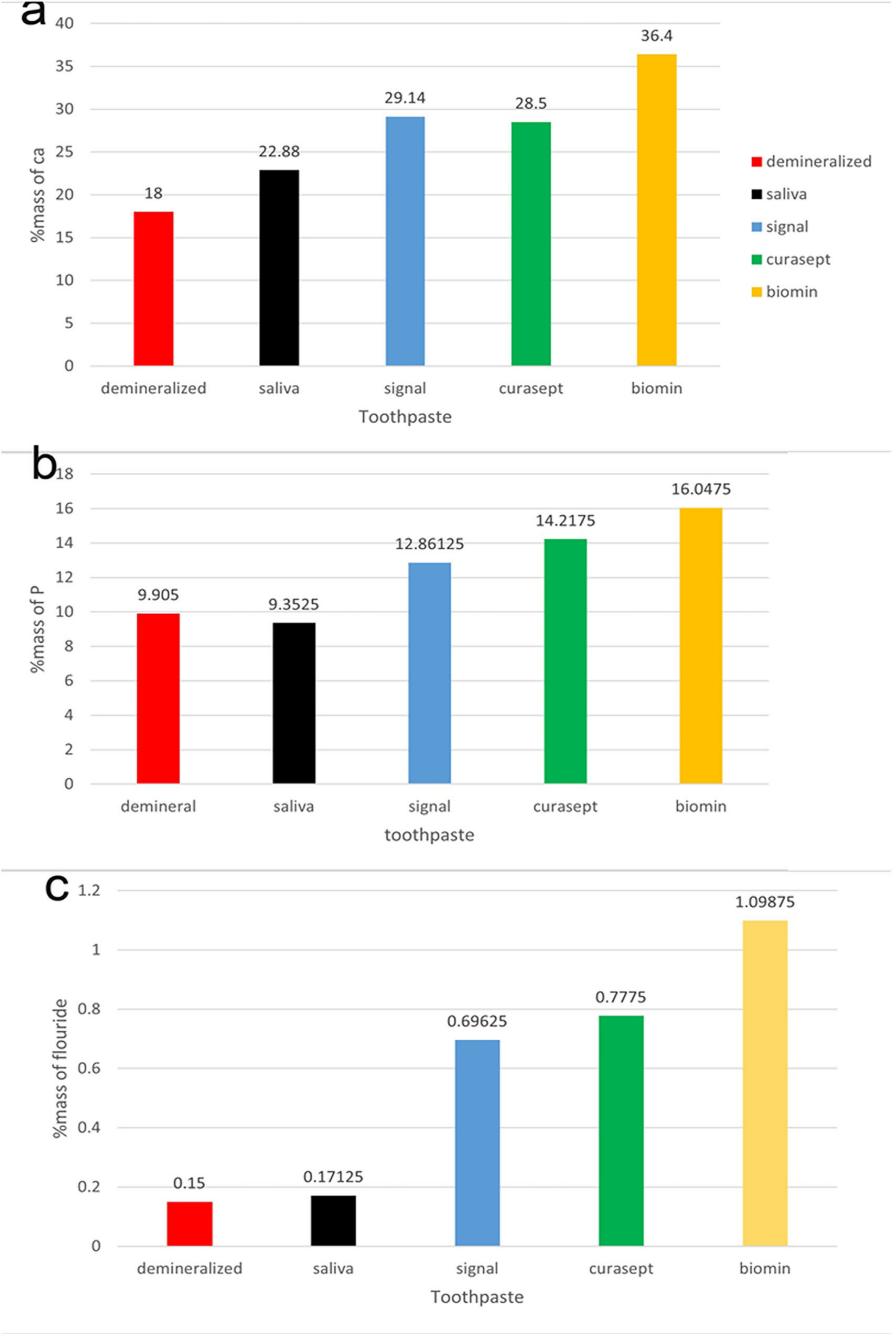
Bar graph showing the mean differences between the five groups. **a** Ca(%mass). **b** P(%mass), **c** F(%mass).

**Fig. 6:**
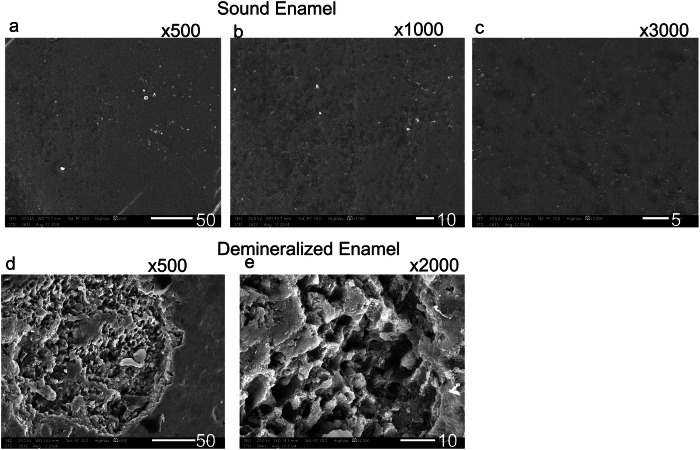
Scanning electron micrograph showing differences between sound and demineralized enamel. Scanning electron micrograph of sound enamel (**a**–**c**) and demineralized enamel (**d**, **e**). At baseline, micrographs (**a**) at *500, (**b**) at *1000, and (**c**) at *3000 magnification showed sound enamel with a smooth surface and minimal visibility of the enamel prisms. The enamel indentations, indicated by arrows, represent the location of the Tomes process in the rodless enamel. After demineralization, micrographs (**d**) at *500 and (**e**) at *1000 (Group 1) revealed areas of dissolution and pores, characterized by a honeycomb appearance. The enamel prisms became more visible due to artificial caries, showing erosion of the enamel rods and partial loss of the interprismatic substances.

**Fig. 7:**
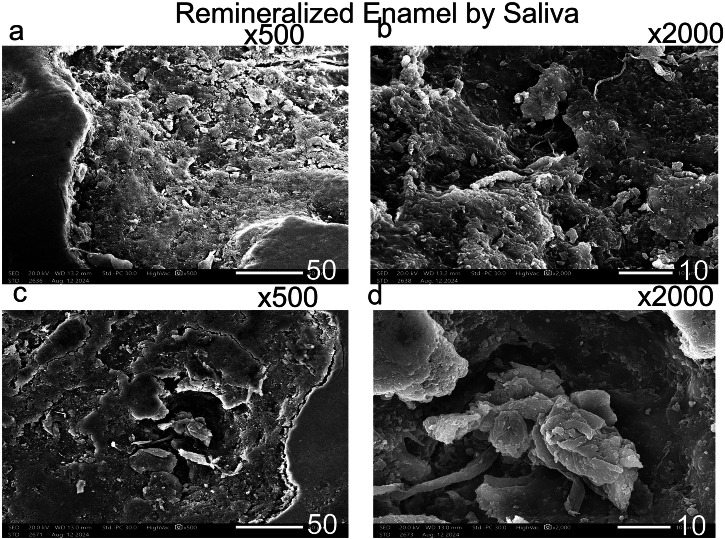
Scanning electron micrograph showing remineralized enamel by saliva. After remineralization by saliva, micrographs (**a**) *500, (**b**) *1000, (**c**) *500, and (**d**) *1000 magnification (Group 2) showed relatively improved mineralization, though it was neither uniform nor homogeneous compared to the other groups (3, 4, 5). A large globular mass was observed within a significant enamel pore (arrow).

**Fig. 8:**
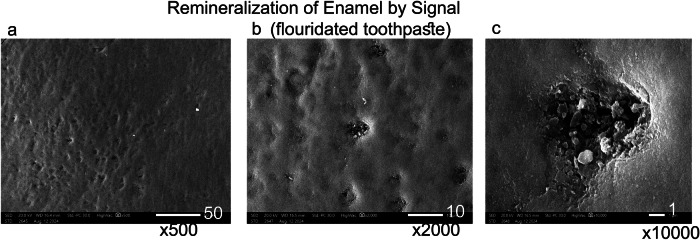
Scanning electron micrograph showing remineralization of enamel by signal. After remineralization with fluoridated toothpaste (Signal 2), micrographs (**a**) at *500, (**b**) at *1000, and (**c**) at *10000 magnification. Group 3 displayed areas of partial restoration of the enamel surface structure, with incomplete filling of voids and porosities caused by the previously induced carious lesion. This indicates the limited remineralization capability of fluoridated toothpaste. Slight roughness, shallow micropores, focal patches, and mild to shallow irregularities were also observed. Small globular masses were noted filling the enamel pores (arrows).

**Fig. 9:**
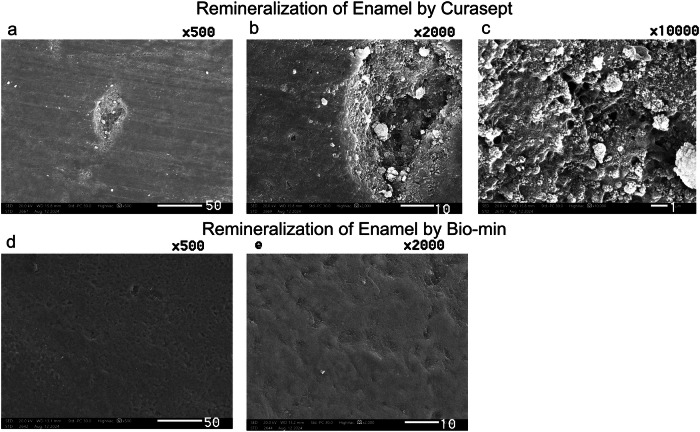
Scanning electron micrograph showing differences between remineralized enamel with Curasept and Biomin. Scanning electron micrograph showing remineralization of enamel by Curasept (**a**–**c**) and by Biomin (**d**, **e**). After remineralization with Curasept toothpaste, micrographs (**a**) at *500, (**b**) at *1000, and (**c**) at *10000 magnification. Group 4 showed a mostly smooth and uniform enamel surface. However, some areas still exhibited remnants of the lesion, which were filled with globular masses(arrow). After remineralization with Biomin toothpaste, micrographs (**d**) at *500 and (**e**) at *1000 magnification. Group 5 revealed significant improvement of the enamel surface, showing complete remineralization and restoration of a smooth enamel surface. The enamel lesion areas had completely disappeared.

3.4 The Scanning electron microscope assessment revealed distinct enamel surface morphologies for each group (Figs. 1–9). The sound enamel exhibited the characteristic regular appearance of an intact surface structure (Fig. 6a, b, c). In Group 1 (G1), the enamel prisms were severely damaged, displaying an irregular surface with heterogeneous pits, grooves, and intense porosity, predominantly in the rod-interrod regions (Fig. 6d, e). Micrographs of Groups (G2) and (G3) showed partial recovery of the enamel structure, with a reduction in both the amount and depth of enamel pores. Mineral deposition was evident through the presence of globular masses on the enamel surface (Figs. 7 and 8). SEM micrographs of Groups (G4) and (G5) demonstrated that the porous structure had transformed into a flatter surface due to remineralization, with a significant reduction in porosity and surface irregularities (Fig. 9). G4 displayed homogeneous remineralization with minimal focal erosions and signs of globular calcification (Fig. 9a, b, c), while G5 showed more homogeneous remineralization and greater recovery of surface integrity compared to G4 (Fig. 9d, e). Those findings support the conclusion that Biomin had the best remineralization effect followed by Curasept.

## Discussion

In the context of preventive and conservative dentistry, there is increasing interest in innovative methods for remineralizing early enamel lesions. Products containing bioavailable calcium, phosphate, and fluoride are commonly used to restore enamel and manage dentin hypersensitivity. This in vitro study aimed to assess and compare the effectiveness of three different remineralizing agents on enamel after acidic exposure. The null hypothesis was rejected based on the results, with Biomin toothpaste being the most effective in enhancing enamel remineralization compared to the others.

The findings showed that the remineralizing agents promoted subsurface mineral recovery on the demineralized enamel surface. In comparison to previous studies [22, 30–33], it is important to highlight that this research is the first to compare those three distinct active ingredients with different fluoride concentrations on primary exfoliated teeth to determine their impact on the remineralization of enamel via the pH cycle model to simulate the oral cavity environment after 28 days.

The study used SEM-EDS to assess enamel surface structure and measure calcium, phosphorus, and fluoride levels as indicators of remineralization. These factors helped evaluate the impact of the tested agents on enamel health [22]. Surface roughness and microhardness are key indicators of enamel remineralization. Lower roughness and higher microhardness are signals of successful restoration of enamel structure and strength [9, 34].

The microhardness and surface roughness tests showed significant differences between all groups, with Biomin toothpaste showing the greatest improvements. However, no significant differences were found between the untreated and the saliva-treated groups.

Elbakry et al. (2024) worked on extracted permanent premolars treated with specific remineralizing agents including Biomin F (1450ppm) for 28 days. They showed that Biomin treatment significantly increases both surface roughness and microhardness. Although this study differs as we used Biomin (580ppm) on primary teeth and employed a pH cycle model which presents a greater challenge for remineralization, both studies showed similar results. This is because the Ca^2+^ and PO_4_^3–^ ions in Biomin nanoparticles are rapidly released and are very sensitive to the slight pH drops, entering deeply to the enamel pores and attracting more Ca^2+^ and PO_4_^3–^ from the remineralizing agents to the surface, aiding in the repair of the micro-cracks and pores in the enamel and restoring the surface to a smooth and hard state [35].

The EDX analysis revealed no significant differences in the mass percentages of calcium, phosphorus, and fluoride between the saliva-treated and untreated groups (*p*-values of 0.3639, 0.4438, and 0.8633) respectively. However, significant differences were found between the fluoridated toothpaste group and the untreated and saliva-treated groups, with *p*-values of less than 0.001 for calcium, phosphorus, and fluoride levels.

This finding was consistent with the SEM results, which showed improved mineralization in both the saliva-treated and fluoridated toothpaste groups. However, the mineralization was neither uniform nor homogeneous in both groups; instead, it displayed globular masses that filled the voids in the enamel. This is because saliva plays a crucial role in enamel remineralization due to its rich in calcium, phosphate, and bicarbonate ions. These components help replenish lost minerals and contribute to restoring enamel strength [3, 36]. Also, Fluoridated toothpaste aids enamel repair by enhancing mineral uptake. Fluoride enhances the precipitation of calcium and phosphate from saliva into weakened enamel, helping to rebuild it. It also encourages the formation of fluorapatite, a more acid-resistant mineral than hydroxyapatite, making the enamel stronger and more resistant to future damage [3, 36].

Curasept showed significant differences in the mass percentages of calcium, phosphate, and fluoride compared to the untreated and saliva-treated groups (*p* < 0.001). However, there are no significant differences between Curasept and fluoridated toothpaste in the mineral percentages (*p* = 0.8485, 0.0710, and 0.5066) respectively. Despite this, Biomin still demonstrated significantly better EDX results than Curasept. In the SEM Curasept displayed homogeneous remineralization with minimal focal erosions and signs of globular calcification.

Vitiello F. et al. (2022) found that treating permanent teeth with F-ACP (Curasept 1450 ppm) yielded similar results to demineralized enamel based on the Ca/P ratio, but this did not match our findings. The difference was attributed to variations in the remineralization techniques, with their study using a 7-day pH cycling process involving 6 h of daily demineralization, whereas our study exposed teeth to 2 h of demineralization daily for a longer period (28 days) giving chance for more crystals formation, resulting in partial enamel remineralization [8].

Tosco V. et al. (2023) used Curasept toothpaste 1450ppm (F-ACP) on permanent teeth for enamel remineralization, implementing a 28-day pH cycling protocol. They found that the EDX results showed increased remineralization in the Curasept-treated enamel compared to untreated groups (*p* < 0.05), also the SEM results were consistent with our findings. This could be due to their use of the same duration for the pH cycling protocol [9].

The effectiveness of Curasept Biosmalto is attributed to its formulation, which includes carbonate, citrate, and bioavailable calcium and phosphate that aid in remineralizing demineralized enamel. The fluoride helps enamel attract and retain these minerals, promoting the formation of fluorapatite. The product may also contain hydroxyapatite nanoparticles that fill micro-defects in the enamel, improving surface smoothness and integrity. Additionally, carbonate stabilizes the ACP, enhancing remineralization by mimicking the natural enamel formation process. Citrate helps solubilize and stabilize calcium and phosphate, facilitating their absorption into the enamel and promoting better integration of the mineralizing agents into the tooth structure, citrate also helps in the penetration of ACP nanoparticles into enamel and dentin, promoting better integration of the mineralizing agents into the tooth structure [9, 37].

The Biomin EDX results demonstrated the most significant increases in the mass percentages of calcium, phosphorus, and fluoride compared to the other groups (*p* < 0.05). This aligned with the enamel surface’s scanning topography, which revealed complete remineralization and restoration of a smooth enamel surface. Bakry AS et al. (2018) found results similar to ours, observing the effect of Biomin paste on white spot lesions in permanent teeth. They applied one-tenth of a gram of BioMinF® powder mixed with phosphoric acid (pH 2.5) to the lesions and kept the teeth in a remineralizing solution for 24 h. Using SEM/EDS analysis, they noted that BioMinF® formed crystal-like structures on the demineralized enamel, thanks to its high calcium and phosphorus content. The study also highlighted the BioMinF® ability to release low levels of fluoride over 12 h, promoting the formation of fluorapatite, a more stable mineral, especially in acidic conditions [38].

Behl et al. (2024) studied the effect of Biomin F (1450 ppm) on permanent teeth using a pH cycle model for 5 days with 5 min in demineralizing solution, followed by twice-daily brushing and then kept in artificial saliva for the rest of the day. They found significant remineralization, as indicated by SEM and EDX, showing a globular structure with scattered calcium particles. In our study, the SEM images demonstrated better results compared to Behl et al., despite having lower fluoride content. This improvement is likely due to the longer treatment duration, which allowed for the prolonged release of fluoride, thereby enhancing remineralization [39].

Eldeeb et al. (2024) treated white spot lesions on permanent teeth with brushing twice daily by Biomin (1450 ppm) for 2 weeks without a pH cycle model. Their EDX results were similar to ours, but their SEM images showed partial restoration of the surface structure while our study showed complete restoration. This difference is attributed to the extended duration of the pH cycle model, which facilitates the release of more fluoride which is quickly released when the pH is slightly decreased. This is because the pH change disrupts the stability of the fluoride-binding structure in Biomin, facilitating a faster release of fluoride [22].

The reason this study achieved better results, even when using primary teeth with lower mineral content than permanent teeth and facing an acidic challenge in the pH cycle model, is the long treatment period. One of the key advantages of bioactive glass is its capacity to gradually release calcium, phosphate, and fluoride ions over an extended period, providing a longer-lasting protective effect. This is enabled by the polymer that reinforces the bond between the calcium in the bioglass and the calcium in the enamel, minimizing the leaching of the bioactive glass material. Moreover, Biomin F contains tiny bioactive glass particles, which help remineralizing agents penetrate subsurface lesions, allowing for deeper penetration of enamel rods with acid-resistant fluorapatite offering more effective, long-lasting protection.

Based on our previous findings, Biomin is significantly superior to Curasept [31]. The bioactive glass can gradually release calcium, phosphate, and fluoride ions over an extended period, providing a longer-lasting protective effect. In contrast, Curasept may require more frequent application and offer a shorter-term effect. Furthermore, Biomin nanoparticles are generally smaller than those in Curasept F-ACP (40-100 nm), which allows them to penetrate enamel more effectively and deliver minerals to micro-defects [40]. Biomin F also contains a higher concentration of phosphate in the form of bioactive glass particles, releasing a significant amount of phosphate over time for efficient remineralization [38, 41].

This study provides evidence that remineralizing toothpaste such as Biomin F in children can be significantly superior to conventional fluoride dentifrice as a caries preventive strategy. Patient-centered treatment and ease of use with fewer dental visits may produce a non-invasive attractive way to deal with early carious lesions for young children [42]. BioMin also could be included in the treatment of children diagnosed with enamel hypoplasia by strengthening the enamel and promoting remineralization, Pediatric dentists can incorporate BioMin toothpaste in the treatment regimen for children after they complete orthodontic treatment. The gradual remineralization effect could reduce the risk of gingivitis and plaque accumulation around orthodontic appliances.

The study’s limitations are that the pH cycling models used in many research studies may not fully replicate the complex biological processes of the oral cavity, such as the presence of natural saliva, pH fluctuations, and chewing forces. In vitro studies often lack a cariogenic biofilm, meaning they don’t account for bacterial interactions, acid production, and biofilm formation. Additionally, natural saliva contains bacteriostatic elements like lysozymes, antibodies, and cariostatic minerals (e.g., magnesium), which aren’t typically present in these models. Moreover, a balanced, nutrient-rich diet can strengthen teeth and promote remineralization. All those factors can affect the remineralization process.

## Conclusion

Biomin (580ppm) outperformed untreated demineralized enamel followed by Curasept and fluoridated toothpaste. Biomin is the top remineralizing agent for early caries lesions, boosting surface microhardness, improving roughness, and increasing calcium, phosphate, and fluoride levels. Biomin releases significant amounts of Ca2+ and PO43- ions, along with a sustained, gradual release of fluoride over an extended period. Its nanoparticles help deliver crystals into deep lesions, promoting the remineralization of early-stage caries, and could also assist in alleviating dentine hypersensitivity. Additional benefits may include a potential reduction in gingivitis. Releasing fluoride over an extended period may help inhibit the growth of harmful bacteria that contribute to gingivitis.

With such results, it seems fair to conclude that incorporating the remineralizing kinds of toothpaste, especially Biomin toothpaste in the oral health care regimen for young children, can enhance the effectiveness of preventive care and address the needs of high-risk groups of patients. Clinicians can offer the long-term oral health of their young patients, promoting enamel health and reducing the need for invasive restorative treatments.

Therefore, future research should prioritize clinical pediatric studies to evaluate the effectiveness of Biomin toothpaste (580ppm) in remineralizing and preventing caries. Furthermore, additional in vitro studies with longer treatment periods are essential, alongside the new fluoride-free remineralizing agents to assess their effects and minimize the potential risk of fluoride toxicity for children. Also, using natural saliva could help improve the in vitro study conditions.

## Data Availability

The datasets used and/or analyzed during the current study are available from the corresponding author upon reasonable request.
